# Improving Pharmaceutical Protein Production in *Oryza sativa*

**DOI:** 10.3390/ijms14058719

**Published:** 2013-04-24

**Authors:** Yu-Chieh Kuo, Chia-Chun Tan, Jung-Ting Ku, Wei-Cho Hsu, Sung-Chieh Su, Chung-An Lu, Li-Fen Huang

**Affiliations:** 1 Graduate School of Biotechnology and Bioengineering, Yuan Ze University, 135 Yuan-Tung Road, Taoyuan 32003, Taiwan; E-Mails: david0925@hotmail.com (Y.-C.K.); smallbu1023@yahoo.com.tw (C.-C.T.); retyku@hotmail.com (J.-T.K.); a6235187@hotmail.com (W.-C.H.); mike36327@hotmail.com (S.-C.S.); 2 Department of Life Sciences, National Central University, 300, Jhongda Rd., Taoyuan 32001, Taiwan; E-Mail: chungan@cc.ncu.edu.tw

**Keywords:** bioreactor, glycosylation, inducible system, *Oryza sativa*, recombinant protein, suspension cells, transgenic plants

## Abstract

Application of plant expression systems in the production of recombinant proteins has several advantages, such as low maintenance cost, absence of human pathogens, and possession of complex post-translational glycosylation capabilities. Plants have been successfully used to produce recombinant cytokines, vaccines, antibodies, and other proteins, and rice (*Oryza sativa*) is a potential plant used as recombinant protein expression system. After successful transformation, transgenic rice cells can be either regenerated into whole plants or grown as cell cultures that can be upscaled into bioreactors. This review summarizes recent advances in the production of different recombinant protein produced in rice and describes their production methods as well as methods to improve protein yield and quality. Glycosylation and its impact in plant development and protein production are discussed, and several methods of improving yield and quality that have not been incorporated in rice expression systems are also proposed. Finally, different bioreactor options are explored and their advantages are analyzed.

## 1. Introduction

Production of recombinant protein using plant expression system first dates back to the 1980s when plants’ genetic transformability has been confirmed [1]. Soon after, human growth hormone and antibody chains were produced using transgenic tobacco and sunflower [2,3]. In 1997, recombinant avidin was produced commercially using transgenic maize, affirming plants’ capabilities to produce recombinant proteins on a large scale [4]. Since then, numerous antibodies, vaccines, cytokines and other proteins have been produced using plant expression systems in the forms of both mature plants and tissue cell cultures [5]. Plants have numerous advantages over other expression systems such as intrinsic safety (plants are free of animal pathogens and viruses, and they can be grown without using animal-derived materials), lower production and capital costs, and glycosylation pattern similar to that of humans [5–7]. Among these advantages, plants’ ability to perform complex post-translational modification is particularly important because it allows for the production of complicated glycoproteins with glycosylation, which plays major roles in the bioactivity and stability of certain proteins [8,9]. Rice is a well-studied model plant whose genome has been decoded, and its transformation technology has been very well developed [10,11]. As the third most produced crop in the world, rice is also considered to be hypoallergenic, making it an excellent host for protein production and even administration [12]. Recent reports indicate that rice is a suitable host for recombinant protein due to its competitive yield of certain recombinant proteins, such as human serum albumin, compared to other plant expression systems such as tobacco cell cultures and potato tubers [13–16]. Suspension cell culture is a widely used method for producing recombinant proteins, and systems upscaled into bioreactors have been utilized commercially [7,17]. Bioreactors have numerous advantages including lower capital and production costs, scalability, safety from viral transmission and other mammalian pathogens [17,18]. They have been widely applied for the production of numerous proteins and are designed to culture and harvest cells in consecutive cycles while continuously renewing nutrient and oxygen within the chamber [19,20]. This review focuses on recombinant proteins that have been successfully produced in rice and summarizes different approaches in improving the yield and quality of the proteins.

## 2. Recombinant Pharmaceuticals in Rice

Foreign genes of interest may be incorporated into plant systems through transient transformation such as agroinfiltration, virus infection, and magnifection [21–23]. These methods allow plants to temporarily express recombinant protein within relatively short amount of time compared to stable, long term transformation, although none of these techniques have been widely applied in rice. Alternatively, long term transformation is achieved by inserting the transgene into the plant’s genome through methods such as Agrobacterium transfection and particle bombardment [11,24]. Callus cells expressing genes of interest may be grown as suspension cell cultures or regenerated into whole plants [13,16]. Whole plant system has advantages such as large scale production and stable storage means in the form of seeds [16]. In addition, direct oral administration of certain pharmaceutical is possible by using certain parts of the plant, although some limitations still exist [5]. On the other hand, suspension cell cultures also have comparative advantages such as more controlled environment, faster cell growth, and potential to be upscaled into bioreactors [17]. In addition, by utilizing a signal peptide, recombinant proteins produced will be secreted out of the cell into the culture medium, removing the need to extract the protein through cell lysis [25]. Rice has been successfully used to produce human serum albumin, vaccine, antibodies, cytokines, and other pharmaceutical proteins [26]. The characteristics, functions, and yields of protein successfully produced using rice expression systems are summarized (Table 1). Furthermore, the biological activity of the recombinant proteins is also examined. If applicable, the yield rate is compared with that of other plant expression systems.

### 2.1. Human Serum Albumin

Human serum albumin (HSA) is an unglycosylated, monomeric protein that plays important roles in osmotic regulation and pH buffering as well as acts as a carrier protein for fatty acids, thyroid hormones, and numerous drugs [42]. There are currently several studied platforms for producing this human protein such as *Escherichia coli*, *Saccharomyces cerevisiae*, *Pichia pastoris*, and numerous plant expression systems, such as tobacco cultures and potato tubers [14,15,43–45]. However, due to several limitations, none of these systems have offered a reliable way of providing biologically active HSA in large quantity. Consequently, limited human plasma remains the most important albumin source but is unable to meet increasing demand. Extensive transfusion exposes recipients to body fluid transferred disease such as Hepatitis B, Syphilis, and HIV [46].

One of the first publications involving producing HSA using rice suspension cell system using an inducible promoter *αAmy3/RAmy3D* reported a maximum yield of 76.4 mg/L of medium after 4 days of sucrose starvation [13]. The expression system utilized a signal peptide and the HSA, along with other proteins, is secreted into the culture medium. There is more than a 6-fold yield advantage compared to HSA produced using tobacco suspension cell, which was 11.88 mg/L [14]. Alternatively, high yield of recombinant HSA has also been produced in rice endosperm. An endosperm-specific promoter, *Gt13a*, and its signal peptide were used to direct expressed protein into protein storage vacuoles, and a maximum yield of 2.75 g/kg of brown rice was obtained [16]. This is a significant yield improvement over production of HSA using potato tubers (0.01 g/kg of fresh weight) and well above the threshold for cost-effective industrial production, which was 0.1 g/kg of fresh weight [15]. The structure of HSA plays a critical role in its biological activity, and the most unique characteristics of the protein, the 17 disulfide bonds as well as the only free SH group Cys34, have been crystallized and extensively studied [47]. It has been shown that recombinant HSA (OsrHSA) produced using a rice expression is structurally equivalent to plasma HSA, and all 17 disulfide bonds have been identified on OsrHSA using X-Ray crystallography [16].

### 2.2. Vaccines

In addition to the advantages of protein production using plant expression systems described above, plant-based pharmaceuticals can also be applied through direct oral administration using specific parts of the plant, eliminating the needs for purification [5]. However, there are still challenges in oral delivery of vaccines produced in plant parts. Oral administration requires about 100 folds of vaccine delivered through direct injection [48]. Endotoxins that exist inside plants, such as solanine in potato tubers, also raise safety concerns, as they cannot be easily removed [49]. In cases where further processing, such as cooking, is required, thermolabile proteins will be degraded in the process, further reducing effective yield [49]. Despite the challenges described, rice remains a competitive candidate for producing and delivering recombinant vaccines for two important reasons. First of all, rice does not contain endotoxins harmful to the human body. Second, localization of recombinant protein into seeds provides ideal storage due to low protease activity [50]. Several vaccine antigens have been successfully produced in recombinant rice. Envelope protein of Japanese encephalitis virus (JEV) has been expressed in rice leaves, and the maximum yield was 1.1–1.9 μg/mg of total protein [27]. To examine the biological activity of the recombinant vaccine, mice were immunized using both *E. coli* derived JEV envelope proteins and recombinant proteins from rice through oral administration. The rice-derived vaccine was able to elicit higher immunoglobin G (IgG) and immunoglobin A (IgA) responses compared to the recombinant protein derived from *E. coli* [27]. Oral administration of *Chlamydophila psittaci* antigen, MOMP, fused to the B subunit of *E. coli* heat-labile enterotoxin (LTB) produced in rice seeds has also successfully induced mouse immunity to mucosal disease [51]. Other vaccines were successfully produced for the intention of oral administration as uncooked rice powder, and biologically activities were examined through animal tests [38,39]. Transgenic rice expressing mouse dominant T cell epitope peptides of *Cry j I* and *Cry j II* allergens of Japanese cedar pollen was able to prevent the development of allergen-specific immunoglobin E (IgE) and immunoglobin G (IgG) responses [38]. Alternatively, transgenic rice expressing a fragment (p45–145) of mite allergen (Der p 1) containing immunodominant human and mouse T cell epitopes successfully reduced the serum levels of allergen-specific IgE and IgG [39]. It is worth noting that differences in mammalian and plant glycosylation have caused immunogenic response in both mice and human, indicating that further modification may be required for recombinant protein produced using rice expression systems [52,53].

### 2.3. Antibodies

Antibodies are serum proteins that bind to target molecules with high specificity and are widely used for prevention, detection, and treatment of diseases. Recombinant antibodies are shown to provide immunization against pathogens and are potential answers to disease, especially with increasing microbial resistance towards antibiotics as well as new pathogens being discovered [54]. Currently, recombinant human cytotoxic T-lymphocyte antigen 4-immunoglobulin (hCTLA4Ig) has been successfully produced in rice suspension cells using *αAmy3/RAmy3D* promoter with maximum yield of 31.4 mg/L in liquid medium [29]. Another antibody, single-chain Fv antibody (ScFvT84.66) under the control of maize *Ubiquitin-1* promoter, has also been expressed in the leaves and calli of transgenic rice, and the yields were 29 μg/g and 3.8 μg/g of fresh weight of leaves and calli, respectively [40,41]. While antibody production in rice has been relatively rare, other antibodies have been successfully produced in other plants. The first recombinant proteins produced in plants were progeny of the cross of two individual transgenic plants, tobacco and sunflower, expressing single immunoglobulin gamma and kappa chains [3]. Antibodies produced in prokaryotic systems often form inclusion bodies, and harsh chemicals must be applied in order to refold the proteins back into their biologically active state [41]. On the other hand, antibodies produced in animal cells are more expensive to maintain and are susceptible to pathogen contamination [55]. In addition to being pathogen-free and capable of correct protein folding, rice also has yield advantages in several other proteins, as mentioned previously. Therefore, it is worth evaluating the possible comparative advantages of producing those antibodies using transgenic rice platform compared to other expression systems.

### 2.4. Cytokines

Cytokines are signaling proteins in intercellular communication and are involved in diverse regulation processes, such as embryogenesis, immune and hematopoietic systems [56]. Due to high production costs, pharmaceutical application of recombinant cytokines is still very limited. Some cytokines that have been successfully produced in rice culture are described below, and their biological activities are examined.

Granulocyte macrophage colony stimulating factor (GM-CSF) is a cytokine used to promote white blood cell proliferation [57]. The first report of human GM-CSF (hGM-CSF) production in rice was in 2003 through suspension cell cultures, and the maximum yield obtained was 129 mg/L [30]. Since then, improvements have been made in rice suspension cell systems producing hGM-CSF by using methods such as humanizing *N*-Glycan structure and increasing yield by 2–3 folds through reduction of endogenous α-amylase expression, co-expression of proteinase inhibitor, and suppression of cellular cysteine proteinase [8,58–61]. The protein is also produced in other expression systems such as rice seeds and tobacco seeds using rice glutelin promoters *Gt1 and Gt3*. The maximum yields were 1.3% of total soluble protein in rice seeds and 0.03% of total soluble protein in tobacco seeds [31,62]. Mouse GM-CSF (mGM-CSF) was successfully produced using rice suspension cells, with yield of 24.6 mg/L of medium [25]. The same glycoprotein was also produced in tobacco leaves, and the yield was 19 μg/g of fresh leaves [37]. The biological activities of recombinant human and mouse GM-CSF produced in rice suspension culture has also been confirmed using GM-CSF dependent TF1 cells and murine myeloblastic leukemia NFS-60 cells, respectively [8,25].

Interleukin-10 (IL-10) is a pleotropic cytokine that plays numerous roles in immunoregulation and inflammation [63]. It has been successfully produced in rice seeds using *Glub-1* promoter and its signal peptide to localize the protein inside seeds exclusively. The product is determined to be unglycosylated and yield of final purified protein was 2 mg per 40 g of rice used (50 μg/g). The biological activity of recombinant IL-10 was confirmed using mouse bone marrow dendritic cells [35]. The protein was also produced inside tobacco leaves, and the yield was 37.0 μg/g of fresh leaves [36].

INF-γ is a class II interferon responsible for regulating immune response against bacteria and tumor [64]. Production of INF-γ has been performed in rice suspension cells using both constitutive maize ubiquitin promoter and inducible rice *αAmy3/RAmy3D* promoter, and the biological activity has been confirmed using human A549 cell line against dengue virus. An α-amylase signal peptide was added to both in order to allow for secretion of recombinant INF-γ into the culture medium. Highest yield obtained from the culturing medium of the ubiquitin promoter driven system was 12 ng/mL and 17.4 ng/mL in *αAmy3/RAmy3D* promoter driven system, and yield found inside the cell was 699.79 ng/g of fresh cell weight for the ubiquitin promoter driven system and 131.6 ng/g of fresh cell weight for the *αAmy3/RAmy3D* promoter driven system [34].

### 2.5. Other Proteins

Human α_1_-antitrypsin (hAAT), a serine trypsin inhibitor, is the most abundant protease inhibitor in human plasma [65]. Its deficiency is known to lead to chronic obstructive pulmonary disease, also known as emphysema [66,67]. Production of human α_1_-antitrypsin in recombinant rice suspension cells was first achieved in 2000, with yield of 120 mg/L, cultured in 30 mL of medium with 10% (*v*/*v*) of cell density [68]. A 350 mL membrane bioreactor system was used to produce human α_1_-antitrypsin, and maximum yield of 247 mg/L was obtained [33]. A larger 5 liter bioreactor was incorporated to producing hAAT, and the maximum yield was 7.3 mg/L [69]. The half-life of recombinant hAAT is shorter than that of plasma hAAT, possibly due to the absence of sialic acid, implying that proper post translational modification will increase the stability of recombinant protein [70].

Human growth hormone (hGH) is responsible for reproduction, growth, and regeneration of certain cells such as muscle and bone cells [71]. It is often prescribed to patients with growth deficiency and is essential for metabolic regulation. Recombinant hGH has been successfully produced in rice suspension cells using *αAmy3/RAmy3D* as a promoter. The maximum yield is 57 mg/L in a culture medium, and the biological activity of the recombinant protein has been confirmed using Nb2 node lymphoma cells, whose proliferation and growth depend on the presence of hGH [32].

A fungal immunemodulatory protein (FIP-*fve*) was isolated from golden needle mushroom (*Flarnmulina velutipes*) and reported to inhibit allergy reactions in mice and regulate Th2 cytokines [72,73]. In addition, it acts as a strong adjuvant that enhanced the immunogenicity of the human papillomavirus type-16 (HPV-16) E7 oncoprotein and conferred increased antitumor effects against the inoculation of HPV-16.E7-expressing TC-1 cells in mice. Recombinant FIP-*fve* fused with the antigen Der p 2 (OsDp2Fve) has been produced using rice suspension cells under a control of *α-amylase* (α*Amy*8) promoter, and the yield was about 7.5 μg/mL (10.5% of total protein) [28].

## 3. Improvement of Rice Expression System

Despite its many advantages, production of recombinant proteins using rice expression systems is still limited by yield, ranging from 12 μg/L to 247 mg/L (Table 1). Several strategies targeting various stages of protein expression have been incorporated to improve the yield. In addition, the significance of presence of and difference in glycosylation are discussed, and different modifications, which have been experimented with, are summarized.

### 3.1. Increase Protein Yields

Physical and chemical approaches have been used to improve protein yield. Currently, rice suspension cells are extensively utilized due to their fast cell doubling time (as fast as 1 day) [7,69]. On the other hand, whole plant systems have been very established, and certain organelles, such as seeds and tubers, act as stable storage locations. Thus, selection of expression system should be based on the protein’s properties, the expression cassette, and purification concerns. In both systems, further yield improvements can be made by modifying promoters of the expression cassettes, the codons of gene responsible for translating the protein, and modifying genes within the host cells.

#### 3.1.1. Promoters

Different promoters have been chosen based on the properties of the protein produced. In terms of constitutive promoters, the *35S* promoter obtained from cauliflower mosaic virus and ubiquitin promoter obtained from maize are most frequently used. The main advantage of constitutive promoters is that they continuously express the gene in all parts of the plant. However, it was demonstrated that spontaneous transgene-silencing phenomena are caused by methylation of the CaMV 35S promoter during transgenic plant growth [74,75]. In addition, there is an increased chance of homology dependent gene silencing (HDGS) occurring. Despite stable integration of *35S:GUS* gene into rice genome, β-glucuronidase (GUS) expression was not found in a transgenic rice line due to HDGS [76]. Alternatively, a number of inducible promoters that only activate during specific conditions or tissue-specific promoters have been chosen in the production of numerous recombinant proteins. While gene HDGS has not been identified in inducible systems, inducible systems are also newer than constitutive systems and not as extensively studied. Therefore, the possibility of gene silencing in inducible systems should be explored. Several induced promoters and their characteristics are discussed below.

Recombinant protein systems using steroid-regulated promoters require only trace amount of steroid to activate the expression system. Expression of green florescent protein (GFP) inside rice calli using estrogen receptor fused chimeric transcription factor, XVE, was studied and compared with *35S:GFP* expression system [77]. GFP expression levels were similar between the constitutive and inducible systems in the roots, while expression level of the inducible system was lower than the constitutive system in leaves. By using different estradiol concentrations (from 1 to 25 μM), GFP expression level peaked at different times and the green fluorescence emitted longer for cells grown on plates with higher estradiol concentration. GFP expression level remains observable for at least one day after calli were moved to an estradiol-free medium [77]. The relationship between the concentration of estradiol present in the medium and the number of days the bioreactor is operated should be explored, as estradiol degradation will lead to a decrease in protein production. In addition, degradation rate of mRNA of steroid induced system should also be examined in order to determine the ideal time for medium exchange or steroid addition.

Ethanol-induced system expressing distinct reporters, chloramphenicol acetyltransferase (CAT) and GUS, in tomatoes was examined [78]. Garoosi *et al.* reported that, under constant presence of 0.1% (*v*/*v*) ethanol, both CAT and GUS activities were detectable within 4 h [78]. GUS expression level of an alcohol-induced system is comparable to that of 35S constitutive promoter. Ethanol should also be maintained under lower concentration, as volume percentage higher than 1% proved to be toxic for plant tissues [78]. In the case of whole-plant production, consistent alcohol concentration may be hard to control due to its volatility. Finally, continuous drenching of root, even in lower concentration ethanol, may have detrimental effects on plant development.

Metabolite-regulated promoters are triggered in the presence or absence of certain metabolic products. *αAmy3/RAmy3D* is one of the most widely used metabolite-regulated promoters and is highly expressed during sugar starvation [30]. In a suspension cell system expressing recombinant HSA using *αAmy3/RAmy3D* promoter, the promoter is activated within 12 h of sugar starvation. Cell viability drops significantly to about 30% within 24 h of sugar starvation. HSA secretion reaches the maximum after four days of sugar starvation, during which cell viability drops nearly to zero. However, once sugar is added into the culture again, cell viability is able to make a full recovery at least for seven cycles, implying that sugar-starvation driven system does not have permanent negative impacts on the cells [13]. Since expression of recombinant protein only takes place under sugar starvation, culture medium exchange must take place in order to maintain cell viability. Lastly, a sugar starvation driven promoter should be sensitive to the absence of sugar so that recombinant proteins can still be produced while cells still have spare resource to produce proteins not essential for its survival.

Production of GUS using the heat-induced *small heat shock protein 18.2* (*sHSP 18.2*) promoter in liquid tobacco root cultures has been performed [79]. Expression level was higher in younger cells and GUS expression level reached maxima when the heat shock treatment is performed at 42 °C for 2 h followed by 27 °C recovery for 24 h. A similar experiment is also performed in transgenic wheat and shows expression throughout the entire plant exposed to heat shock treatment, although the expression level is lower in proximal roots than in root tips [80]. Constant cycles of heat shocks and recovery may cause irreversible effects on cell metabolism, which may lower cell viability. Furthermore, the property of recombinant protein under constant temperature fluctuation should also be studied before using a temperature-regulated expression system. Finally, additional energy is required to perform heating, which should also be taken into consideration during commercialization.

#### 3.1.2. Rice Optimized Codon Usage

The preferred codons vary significantly between different plants, so when the desired foreign gene contains rare codons the host plant, the codon becomes a limiting factor in protein translation [81,82]. This is a major drawback in the goal of mass production of recombinant proteins in plants. To overcome this limitation, a plausible strategy involves increasing translation efficiency through modifications of codons from the original DNA sequence to more suitable ones in the host plant without changing the amino acid sequence [83]. In plant expression systems, expression of *Bacillus thuringiensis cryIA(b)* gene, primarily used to genetically increase tomatoes resistance towards insects, was increased 100 folds by making 21% changes to the original *cryIA(b)* DNA sequence [84]. Production of miraculin, a taste-altering glycoprotein, in transgenic tomato is also improved by codon optimization, and yield improved from 196.5 to 286.9 μg/g of fresh weight [81]. Codon modification was also performed in rice to express phytase of *Saccyromyces cerevisae*, and phytase activity in rice leaves increased from 0.039 U/g of fresh leaves of transgenic rice using unchanged codon to 4.6 U/g using full-length modified codons and 10.6 U/g using truncated codons (U is defined as 1 μmol phosphate released per min at 37 °C) [85]. By combining codon optimization with the usage of KDEL sequence, expression of adioponectin increased from 0.06% to 0.32% of total soluble protein in rice leaves [86].

#### 3.1.3. Endogenous Gene Modification

Modification within the host’s genes is also made in order to increase protein productivity through either reduction of protease expression or alleviation of internal resource competition and feedback regulation. The first strategy used involves knocking down genes expressing endogenous secreted protease such as cysteine proteinase [60]. Results here show up to 1.9 fold of human GM-CSF (hGM-CSF) yield improvement, from 150.4 to 289.1 mg/L. In another case studied, expression of endogenous α-amylase has been knocked down through RNAi [59]. Results indicate that the production of hGM-CSF using *αAmy3/RAmy3D* promoter has almost doubled from approximately 150 to 280 mg/L. Alternatively, studies have also been performed in *Nicotiana benthamiana* in suppressing transcriptional silencing that is responsible for decreased expression level of recombinant protein. Yield of murine IgG was 10.8 mg per gram of leaf fresh weight for normal transgenic line while yield increased 14-fold to 147.7 mg/g of fresh weight of leaves for transgenic line also containing p19, a virus suppressor found in the tomato bushy stunt virus that is able to interfere with plant’s endogenous gene silencing [87,88].

### 3.2. Humanized Glycosylation

One of the biggest challenges for plant expression system remains the differences that exist between mammalian glycosylation and plant glycosylation. In addition to affecting the biological activity of glycoproteins, there are two additional concerns involved with difference in glycosylation. First, human body might recognize plant-specific glycosylation and trigger allergic reactions [89,90]. However, lack of proper glycosylation from normally glycosylated proteins may also induce immune response, as recombinant material produced in *E. coli* that lacks glycosylation showed increased immunogenicity in patients [91]. Furthermore, glycosylation is also known to improve the stability of plasma glycoprotein such as CC chemokine ligand 2 inside the blood through proper folding and increased conformation stability [92,93].

There are several approaches in modifying the post translational glycosylation system and increasing the similarity between recombinant protein produced in plants and their native forms. One way is by knocking down the expression levels of enzymes, α-1,3-fucosyltransferase and β-1,2-xylosyltransferase, responsible for adding plant specific glycans, fucose and xylose respectively. Current study indicates that knockdown of α-1,3-fucosyltransferase and β-1,2-xylosyltransferase in rice does not have negative effects on the functions of the transgenic suspension cell culture in areas such as cell division, proliferation, and ability to secret proteins out of the cell, while using sucrose as the carbon source [58]. However, it has not been determined whether using alternative carbon sources, such as starch, will affect the growth of the transgenic rice cell line. Rice α-amylase, the major enzyme responsible for breaking down starch, is a glycoprotein [94]. Therefore, its biological activity may be tied to its glycosylation. Thus, it is worth investigating whether incomplete glycosylation caused by RNA interference will affect the biological activity of α-amylase in rice. The physiology of the plants should also be studied and compared to wild type plants. For example, deficiency or absence of α-mannosidase I, located in both the ER and Golgi, has negative impacts such as the formation of aberrant *N*-glycans, short and swollen roots, and malformation and function loss of cell walls [95]. Knockdown of two xylosyltransferases has been performed in *Arabidopsis thaliana*, and the transgenic plant possessed aberrant root hairs and lacked detectable xylogulcan [96]. These evidences indicate that knockdown or knockout of the gene involved in *N*-glycosylation may lead to abnormal cell growth and compromise protein production. Consequently, it is suggested that RNA interference should be performed using an inducible system in order to preserve normal plant physiology and express glycosylation change only in proper environment.

Due the importance of high-mannose glycosylation taking place in the ER, another method involves adding the sequence KDEL (Lysine-Aspartic acid-Glutamic acid-Leucine) to the sequence of the recombinant. KDEL is an amino acid sequence capable of performing ER-retention, which prevents the protein from leaving the endoplasmic reticulum (ER) into the Golgi apparatus. Addition of KDEL tag to recombinant protein is rather significant because both plant-specific xylosyltransferase and fucosyltransferase are located inside the Golgi apparatus, and plant-exclusive glycosylation of the recombinant protein would be impossible to perform if the protein is retained inside the ER. Using this method, recombinant human interleukin 10 has been successfully produced from transgenic rice seeds, and the proteins were shown to be unglycosylated and identical to its natural human counterpart, confirming that the addition of the KDEL sequence does not affect the biological activity of the amino acid [35]. In *Nicotiana tabacum*, cPIPP, a mouse/human chimeric antibody specific for the β-subunit of human chorionic gonadotropin, was produced with a KDEL tag [97]. The glycoprotein localized inside the ER does not contain any of its natural plant-specific β-1,2-xylose and α-1,3-fucose glycans [97]. The stability of recombinant protein retained inside the ER *in vivo* also does not appear to differ from recombinant protein produced without a KDEL tag, further indicating removal of plant-specific β-1,2-xylose and α-1,3-fucose can be performed to reduce risk of immunogenic responses without compromising protein stability [98]. The biggest disadvantage of adding an ER-retaining tag to the recombinant protein is that cell lysis will be necessary to recover the recombinant protein, as KDEL also prevents its tagged protein from migrating elsewhere, making protein secretion an ineffective strategy. Consequently, additional protein purification steps will be required to isolate protein from other cell content, especially protease from other organelles that will compromise the stability of the recombinant protein.

### 3.3. Genome Stabilization during Rice Cell Culture

While a liquid suspension cell culture significantly improves cell growth rate and allows for recombinant protein secretion through the application of signal peptides, there are still potential concerns involved with the genome integrity of the transgenic plants. The activity level of retrotransposon inside undifferentiated rice suspension cultures is relatively high compared to differentiated cells, partially due to the high speed of replication. Retrotransposons are Class I transposable elements (TE) that are capable of performing reverse transcription using self-encoded reverse transcriptase, and the RNA fragments created will be integrated back into the genome [99,100]. Consequently, the increased number and changing locations of the retrotransposon could have a potential impact on the genomic stability of the host cells, which is a concern in maintaining cell lines expressing recombinant proteins. Thus, the changing level of transposons within cell lines over the course of time is an important indicator. It was observed that in rice cultures containing *Tos17* activity, the expression level of the retrotransposon remained high and increased over the course of one year [101].

To preserve genome integrity and limit activity of retrotranspon, it may be advisable to keep unused cells under cryopreservation [102]. Arabidopsis cell lines have been cryogenically frozen at −30 °C or −80 °C and then thawed out, and the cell activity remained similar to unfrozen cells [103]. A similar experiment was also performed in transgenic rice expressing recombinant hCTLA4Ig and cells recovered from cryopreservation banking had cell viability of 88% [102]. These experiments indicate that cryopreservation banking is a useful tool in conserving transgenic rice cell lines under controlled low temperature, and better temperature parameter for both freezing and thawing should be experimented for optimal rice cell viability recovery. It may also be appropriate to store transgenic cell lines capable of producing recombinant proteins in the form of rice grains, as cell division stops at this period. Seeds can be grown into whole plants or induced into cell cultures using auxin such as 2,4-Dichlorophenoxyacetic acid if they are needed for protein production in the future.

## 4. Large Scale Production

The methods proposed above are used in rice or other plant expression systems to improve average yield per cell. In order to further increase recombinant protein production, plant suspension cell cultures should be upscaled into bioreactors. Large-scale bioreactor producing plant secondary metabolite has already been well established [104]. By using a bioreactor, it is possible for plant production of recombinant protein to increase to a level similar to mammalian expression systems [7]. In 2010, Protalix has developed a high-volume bioreactor (up to 75,000 liters) using carrot as a host organism to produce taliglucerase alfa (http://www.protalix.com/index.asp), a plant-derived enzyme used to treat patients with Gaucher’s Disease [105]. The pharmaceutical became the first plant pharmaceutical to be approved by the FDA on 1 May, 2012. Efforts are also made in disposable bioreactors capable of meeting GMP standards [106]. However, there is very limited number of bioreactor production of pharmaceutical using rice cells. While overall protein production increases, average protein production per cell actually may decrease in bioreactors, possibly due to compromised environment and increased endotoxic materials, secondary metabolite, and feedback regulation. For example, production of human α_1_-antitrypsin reached 247 mg/L in a 300 mL membrane bioreactor, but dropped down to 7.3 mg/L when upscaled to a 5 liter bioreactor [33,69]. Furthermore, the biggest challenges in bioreactor upscaling are the rheological and hydrodynamic shearing [107], and plant cells are sensitive towards shearing due to inflexible cellulose-based cell wall [7]. Cell aggregate is advantageous in some situations, as increase in sedimentation rate allows media exchange and culture broth recovery become easier to perform. However, increase in cell aggregate makes cells more susceptible to shearing and can cause uneven distribution of oxygen and other nutrients within larger aggregates [108]. A few examples of rice bioreactors are described, and potential new options are proposed below.

Fed-batch reactors involve the addition of nutrient and cells at the start of cultivation or at certain points during the reaction process. The main advantage of the system is its relatively easy implementation. However, the system suffers major drawbacks such as depletion of major nutrients and accumulation of potentially inhibitory metabolites. Furthermore, as the concentration of product increases inside the medium, feedback regulation may take place to limit further production of the recombinant protein. Cell growth also slows down at higher cell density. Cleanup of the system between usages is also very laborious and time consuming. Regardless, batch-fed bioreactors have been successfully utilized to produce hCTLA4Ig and the maximum yield obtained was 76.5 mg/L. A sugar-starvation driven *αAmy3/RAmy3D* promoter was used here and protein yield was 1.2 times higher than when a constitutive promoter was used, indicating that conditions can be changed to optimize protein production [109]. Perfusion and continuous cultures may also be used to refresh nutrient and remove product as well as metabolites from the bioreactor, and excessive cells can also be removed in order to maintain an ideal growth condition similar to when the bioreactor was first applied.

Current application of rice bioreactor producing recombinant protein with the highest yield utilized a membrane bioreactor [33]. A membrane with 50 kDa molecular weight cut off (MWCO) was suspended inside the bioreactor and used to exchange nutrient and waste liquid. Another gas exchange membrane is located near the bottom of the bioreactor and is used to remove CO_2_ and introduce O_2_ into the system. The yield of recombinant human alpha-1-antitrypsin in this system was 247 mg/L. The biggest limitation of a membrane based system involves the cleanup of membranes as cell aggregates begin to adsorb on the membrane, as the system usually needs to be paused in order for the cell mass to be removed. While continuous scraping is a viable way of removing debris from the membrane in bioreactors used to treat waste water, care must be taken when applying the same system to plant recombinant protein bioreactors. Disposable membranes are recommended in this case.

In addition to protein yield decrease due to depleting nutrients and accumulating products that may contribute to feedback regulation, long-term bioreactors also have several other challenges, including the time, labor, and cost involved in cleaning the bioreactor containers. Furthermore, loss due to contamination is much more significant when small numbers of large volume bioreactors are used compared to larger numbers of smaller bioreactors. Thus, disposable, smaller volume, bioreactors using plastic containers have become increasingly more popular in animal suspension cells [110]. In addition, there have been studies indicating that protein loss involved in suspension cell cultures may be associated with adsorptive properties of glassware, indicating that material used as bioreactor tank may also play important roles in maximizing protein yield [111]. An air-lift bioreactor using a 5-liter plastic container has been utilized to produce mouse GM-CSF in rice cells [25]. While large volume bioreactors may have advantages in economies of scale, it may be less risky and practical to incorporate smaller bioreactors in large numbers to produce certain proteins.

## 5. Improvements in Downstream Processing

Purification cost of plant-derived pharmaceuticals is estimated to account for 80% of total cost [112]. Therefore, reducing the number of purification steps required will greatly reduce the cost associated with protein production. In most of the rice suspension cells system described above, a signal peptide is usually used to guide the protein into the growth medium, eliminating the need for cell lysis. A new idea using the application of oleosin in protein separation has been used in seeds of plants with high oil body contents. Expression system of target protein fused with oleosin has been performed in safflower to produce Apolipoprotein AI (Milano) [113]. Human insulin-like growth factor 1 and insulin have also been produced using oleosin fusion technology inside Arabidopsis [114,115]. While rice is not rich in oil content, it is worth testing whether oleosin fusion protein can be localized in oil-rich section of rice cell or if the oleosin fusion protein can be secreted into the culture medium. Alternatively, if oleosin fusion proteins can be properly secreted out of the cell into the medium using signal peptide to targeting out of the cell into medium, this system may still be useful in purifying recombinant proteins produced in rice.

## 6. Conclusion and Future Remarks

Development of plant-based recombinant protein expression system has greatly improved protein yield in plants. The incorporation of both transient and stable transformation techniques has allowed for either fast recombinant protein expression or the establishment of transgenic cell cultures [5,7]. Whole plant systems are safe, competitive candidates for producing pharmaceuticals and have niche roles in oral administration, although some challenges exist [8,45]. Alternatively, suspension cells can be grown in isolated environments with controlled medium and hormone supply, and cell division time is shortened to 1–1.5 days [69]. Further upscaling to bioreactor allows for even higher protein production. In addition to trying to further improve protein yield and production cost effectiveness, focus should be also placed on humanizing the proteins through glycosylation modification and further improving protein stability and purification processes. Furthermore, as evident by rice’s excellent production yield mentioned earlier, it will also be worthwhile to develop and optimize bioreactors for rice. It is projected that four of five top-selling drugs will be therapeutic proteins by 2013 [116], and through further improvement of expression systems and genome stabilization, coupled with existing advantages such as high yield, lack of human pathogens, and complex existing glycosylation systems, rice cells will be an ideal host for producing protein therapeutics.

## Figures and Tables

**Table 1:** Pharmaceuticals produced in rice and yield comparison to other plants.

Product	Plant	Promoter	Localization	Maximum Yield	Reference
Envelope protein of Japanese encephalitis virus (JEV)	Rice	*CaMV 35S*	Leaf	1.9 μg/mg *	[27]
FIP-*fve* fused with the antigen Der p 2 (OsDp2Fve)	Rice	*αAmy8*	Cell culture medium	7.5 mg/L	[28]
Human cytotoxic T-lymphocyte antigen 4-immuno globulin	Rice	*RAmy3D*	Cell culture medium	31.4 mg/L	[29]
Human granulo cyte macrophage-colony stimulating factor (hGM-CSF)	Rice	*Ramy3D*	Cell culture medium	129 mg/L	[30]
Human granulo cyte macrophage-colony stimulating factor (hGM-CSF)	Tobacco	*Gt1/Gt3*	Seed	0.03% TSP **	[31]
Human growth hormone (hGH)	Rice	*RAmy3D*	Cell culture medium	57 mg/L	[32]
Human serum albumin	Rice	*RAmy3D*	Cell culture medium	76.4 mg/L	[13]
Human serum albumin	Tobacco	*CaMV35S*	Cell culture medium	11.88 mg/L	[14]
Human serum albumin	Potato	*B33*	Tuber	0.01 μg/g *	[15]
Human serum albumin	Rice	*Gt13a*	Seed	2.75 g/kg *	[16]
Human α_1_–antitrypsin (hAAT)	Rice	*Ramy3D*	Cell culture medium	247 mg/L	[33]
Interferon Gamma (INF-γ)	Rice	*Ubi-1*	Cell culture medium	12 ng/mL	[34]
Interferon Gamma (INF-γ)	Rice	*RAmy3D*	Cell culture medium	17.4 ng/mL	[34]
Interleukin-10 (IL-10)	Rice	*Glub1*	Seed	50 μg/g *	[35]
Interleukin-10 (IL-10)	Tobacco	*CaMV 35S*	Leaf	37.0 μg/g *	[36]
Mouse granulo cyte macrophage-colony stimulating factor (mGM-CSF)	Rice	*RAmy3D*	Cell culture medium	24.6 mg/L	[25]
Mouse granulo cyte macrophage-colony stimulating factor (mGM-CSF)	Tobacco	*RbcS1*	Leaf	19.9 μg/g *	[37]
*Cry j I*, *Cry j II*	Rice	*Glub1*	Seed	7 μg/rice grain *	[38]
*Der p I*	Rice	*Glub1*	Seed	7.5 μg/rice grain *	[39]
Single-chain Fv antibody (ScFvT84.66)	Rice	*Ubi-1*	Leaf	29 μg/g *	[40]
1	Rice	*Ubi-1*	Seed	3.8 μg/g *	[41]

* Fresh weights indicated.

** TSP: Total soluble protein. Cell culture medium localization indicates that recombinant protein was secreted via signal peptide into the culture medium. The medium is then collected and no cell lysis is performed.
